# Neurocognitive profile in major depressive disorders: relationship to symptom level and subjective memory complaints

**DOI:** 10.1186/s12888-016-0815-8

**Published:** 2016-04-19

**Authors:** Christine Mohn, Bjørn Rishovd Rund

**Affiliations:** Research Department, Vestre Viken Hospital Trust, Wergelands gate 10, 3004 Drammen, Norway; Department of Psychology, University of Oslo, Oslo, Norway

**Keywords:** Cognition, Depression, MCCB, Memory, Neuropsychology

## Abstract

**Background:**

The MATRICS Consensus Cognitive Battery (MCCB) was developed for schizophrenia patients, but is also being used to assess neurocognitive function in bipolar disorder. This study aims to describe neurocognitive differences in major depressive disorder patients and healthy controls with the MCCB, and to describe the relationship between depression symptom severity, subjective cognitive complaints, and objective cognitive test performance.

**Methods:**

Thirty-three patients with major depressive disorder and 33 pairwise matched healthy controls were assessed with the MCCB. The patients were also assessed with the Montgomery-Åsberg Depression Rating Scale (MADRS) and the Everyday Memory Questionnaire (EMQ).

**Results:**

On all neurocognitive domains, the depression patients scored significantly lower than the controls. The level of impairment ranged from 21.0 % (Working Memory) to 58.0 % (Speed of Processing). There were significant associations between neurocognitive test performance and depression symptom severity, but not with subjective cognitive complaints.

**Conclusions:**

The MCCB was applicable in this study of major depressive disorder, and revealed significant neurocognitive dysfunction in this group. At least one fifth of the patients were impaired on all cognitive domains, with Speed of Processing and Reasoning/Problem Solving being most strongly affected. The objective test scores were significantly related to depression severity, but not to subjective cognitive complaints.

**Electronic supplementary material:**

The online version of this article (doi:10.1186/s12888-016-0815-8) contains supplementary material, which is available to authorized users.

## Background

Mild to moderate cognitive dysfunction is a common symptom in major depressive disorders (MDD). Speed of processing, executive function, working memory, and learning/short-term memory seem to be more strongly affected than other functions [1–3]. Although some differences between acute and remitted states have been noted [1], mild cognitive impairment may be present in recovered individuals as well [4].

The MATRICS Consensus Cognitive Battery (MCCB) [5] is one of the most popular assessment batteries for neurocognitive function in severe psychopathology. It consists of 10 tests assessing 7 cognitive domains – Speed of Processing, Attention/Vigilance, Working Memory, Verbal Learning, Visual Learning, Reasoning/Problem Solving, and Social Cognition. It was developed for the schizophrenia population, but is also used with bipolar disorder patients [6–9], and recently with MDD patients [10]. Using a comprehensive battery consisting of highly reliable and valid tests selected with respect to their tolerability in severely ill patients will facilitate comparison across studies.

Studies comparing neurocognitive function, using tests that assess similar domains as the MCCB does, have found cognitive impairment in both schizophrenia and depression disorders, but the level of impairment is more severe in the former group [11–13]. In these studies, speed based tests scores and verbal memory functions were most strongly affected. A similar pattern of results was obtained in a recent study of MCCB performance in bipolar disorders [7]. Moreover, this study demonstrated that the Norwegian version of the MCCB is well tolerated and applicable in mood disorders [7].

As studies with the MCCB in other groups than schizophrenia patients are scarce, there is little information on the relationship between depression symptom severity and test performance. In the bipolar disorder study of Kessler et al. [7] symptom severity was not correlated with MCCB test performance. A similar finding of no significant association in depression patients tested with a battery that roughly corresponds to the MCCB was reported by Rund et al. [13]. However, processing speed alone has been found to correlate with depression symptom load [14]. We aim to study the same associations using the MCCB in an MDD sample.

There is a well-known discrepancy between subjective cognitive complaints (SCC) and objective test performance, both in individuals suffering from mental illness [15] and healthy individuals [16, 17]. Usually, a person may experience concentration problems and memory lapses during work or activities of daily life, but still perform adequately during neuropsychological assessment. For individuals suffering from MDD, this discrepancy may be partly explained by the severity of the depressive symptoms, as depression intensity is correlated with SCC [18, 19]. In this study, we will describe the relationship between SCC and MCCB performance.

Electroconvulsive therapy (ECT) for treatment-resistant depression often results in a rapid decrease in symptoms. However, a sizable minority of patients report reduced cognitive function, at least during the first weeks after treatment [20]. Despite cognitive side effects in some patients, or fear of such in others, monitoring of post-treatment cognitive function is not routinely performed [21]. The lack of systematic and longitudinal monitoring has made it impossible to ascertain how long the cognitive side effects of ECT last. This paper is the first issued from a 2-year longitudinal project on cognitive effects of electroconvulsive treatment (ECT) for MDD in South-Eastern Norway.

In the current paper, baseline neurocognitive function in MDD is the main topic. Specifically, we aimed to study neurocognitive differences in MDD and healthy controls, and to describe the relationship between depression symptom severity, subjective cognitive complaints, and objective cognitive test performance.

## Methods

### Participants

Demographic data are presented in Table 1. The patient group consisted of 33 participants with a major depressive disorder recruited from the ECT clinical sections at Vestre Viken Hospital Trust. All patients set to undergo ECT and fulfilling the inclusion criteria of the project were invited to participate. Less than ten eligible patients declined. The patients were included from March 2011 to November 2014.

**Table 1:** Demographic characteristics of the participants

	Depression group (*n* = 33)	Control group (*n* = 33)
Age	46.5 (10.6)	46.7 (10.9)
Gender	10 males, 23 females	10 males, 23 females
Education		
Elementary school	*n* = 10 (30.3 %)	*n* = 8 (24.2 %)
High school	*n* = 12 (36.4 %)	*n* = 15 (45.5)
BA/BA +	*n* = 11 (33.3 %)	*n* = 10 (30.3 %)

Age in mean years (SD)

All patients were neurocognitively assessed 1–3 days before the onset of ECT. Inclusion criteria were age above 18 and below 70 years, capacity for giving informed consent to both ECT and participation in this project, ability to understand spoken and written Norwegian, and a diagnosis of major depression episode resistant to other treatment methods. Exclusion criteria were ongoing alcohol or drug abuse, previous or ongoing neurological damage or illness, and ECT within the last 2 years.

The diagnosis of a major depression episode was established by clinical interviews by hospital staff according to the DSM-IV criteria [22] and supported by information from hospital records. Severity of depression was assessed with the Montgomery-Åsberg Depression Rating Scale [23]. Twenty-three of the patients were diagnosed with recurrent unipolar depression and 10 with bipolar disorder type II. Seven had experienced psychotic symptoms during depressive episodes, nine had moderate to severe anxiety symptoms, and four partially fulfilled the criteria for a personality disorder. Five of the patients had been treated with 1–2 series of ECT more than 2 years previously. All patients had discontinued their psychotropic medication 1–7 days before testing, although medication for anxiety and insomnia had been permitted the evening before. Two patients did not use any regular medication at inclusion. See Table 2 for information on the daily doses of medication [24].

**Table 2:** Clinical characteristic of the depression group (*n* = 33)

Years since first onset of depression	18.4 (SD 11.2, range 3–40)
MADRS score	33.5 (SD 7.7)
EMQ score	104.0 (SD 37.8)
Medication (CDD)	
Antidepressants	1.4
Antipsychotics	0.6
Lithium	0.8
Anticonvulsants	0.6

Years since onset, MADRS score, and EMQ score in mean. CDD: Calculated dose of medication based on the prescribed dosage divided by the defined daily dosage

The control group consisted of 33 healthy men and women pairwise matched with the patient group on gender, age, and level of education. The recruitment and assessment procedure of the healthy controls have been described in detail elsewhere [25, 26].

All participants signed an informed consent form before testing. This study was approved by the Regional Committee for Research Ethics for Health Region South-East (REK Sør-Øst).

### Neuropsychological assessment

The cognitive assessment was carried out by a clinical psychologist with extensive neuropsychological training (CM). The patients were tested at their respective clinical wards, and the control group at the University of Oslo or at the facilities of Vestre Viken Hospital Trust.

All participants were tested with the MCCB, covering the following seven domains [5, 27]: Speed of Processing (Trail Making Test A; TMT-A [28], Symbol Coding; Brief Assessment of Cognition in Schizophrenia, BACS [29], and Fluency; Category Fluency [30]), Attention/Vigilance (The Continuous Performance Test-Identical Pairs; CPT-IP [31]), Working Memory (Spatial Span; The Wechsler Memory Scale, SS-WMS [32] and Letter Number Span; The University of Maryland Letter Number Span test, LNS [33]), Verbal Learning (the revised Hopkins Verbal Learning Test; HVLT-R, immediate recall [34]), Visual Learning (the revised Brief Visuospatial Memory Test; BVMT-R [35]), Reasoning/Problem Solving (The Mazes test; Neuropsychological Assessment Battery, NAB [36]), and Social Cognition (the Managing Emotions part of the Mayer-Salovey-Caruso Emotional Intelligence Test; MSCEIT [37]). In addition, a Composite sum score is calculated across the seven domains.

As there are no norms for the MCCB scores of tests respondents above the age of 59, our results are reported as T scores with a mean of 50 and an SD of 10. Due to excessive fatigue, three of the patients did not perform the MSCEIT test and nine of them did not perform the CPT-IP test. This is indicated in Tables 3 and 4.

**Table 3:** Neurocognitive scores of the MCCB (T scores) in depression patients (*n* = 24–33) and healthy controls (*n* = 33)

	Depression	Controls	F	η^2^
Speed of Processing	45.4 (8.5)	54.6 (4.9)	28.51 ***	.31
TMT-A	45.2 (11.5)	54.8 (4.6)	20.07 ***	.24
BACS	44.4 (9.2)	55.6 (7.4)	29.14 ***	.31
Fluency	46.7 (11.1)	53.3 (7.5)	8.11 **	.11
Attention/Vigilance (CPT-IP)	46.9 (10.3) (*n* = 24)	52.3 (9.3)	4.29 *	.07
Working Memory	46.9 (8.8)	53.1 (7.0)	10.24 **	.14
SS-WMS	46.2 (10.1)	53.8 (8.4)	11.20 ***	.15
LNS	47.6 (11.5)	52.4 (7.6)	4.04 *	.06
Verbal Learning (HVLT-R)	47.2 (10.2)	54.8 (9.1)	5.47 *	.08
Visual Learning (BVMT-R)	47.1 (11.2)	52.9 (7.8)	6.18 *	.09
Reasoning/Problem Solving (Mazes)	45.6 (11.1)	54.4 (6.2)	15.73 ***	.20
Social Cognition (MSCEIT)	46.9 (10.3) (*n* = 30)	53.4 (9.2)	8.83 ***	.13
Composite Score	48.4 (5.4) (*n* = 24)	53.6 (4.2)	16.30 ***	.23

Neurocognitive scores in mean (SD). F: Significance test of group differences. ***: *p* < .001, **: *p* < .01, *: *p* < .05. η^2^ : effect size

**Table 4:** Correlations of the relationship between depression severity and cognitive variables in the depression group (*n* = 24–33)

	EMQ	TMT-A	BACS	Fluency	SS-WMS	LNS	HVLT-R	BVMT-R	Mazes	MSCEIT	CPT-IP
MADRS	-.33	-.19	-.38 *	-.48 **	-.24	-.50 **	-.52 **	-.37 *	-.01	-.47 **	-.17

**: *p* < .01, *: *p* < .05 (2-tailed)

After the completion of the MCCB, the patients filled in the Everyday Memory Questionnaire (EMQ) [38], assessing practical attention and memory functions in 28 items.

### Statistics

All statistical analyses were performed with IBM SpSS Statistics version 22. Group differences in neurocognitive function were analyzed with ANOVAs with effect sizes reported as partial eta squared (*η*^*2*^). In the patient group, the relationships between the MADRS score, the EMQ score and the cognitive function scores were studied with Pearson’s correlations.

## Results

Compared to the control group, the depression group performed significantly worse on all neurocognitive tests. The effect sizes were largest for the assessments of Speed of Processing and Reasoning/Problem solving, with the patient group performing at a level 1.5–2.0 *SD*s below the control mean (Table 3).

We chose a cut-off point of 1.5 *SD* below the control mean as a sign of impairment. The same cut-off point was chosen by Kessler et al. [7], facilitating the comparison of these two studies of the Norwegian MCCB in depression disorders. When using this cutoff-point, the level of impairment among the depression patients were as follows: Speed of Processing: 58.0 %, Attention/Vigilance: 25.0 %, Working Memory: 21.0 %, Verbal Learning: 24.0 %, Visual Learning: 27.0 %, Reasoning/Problem Solving: 52.0 %, and Social Cognition: 27.0 %.

In order to control for the possible influence of bipolar disorder, anxiety disorders, and previous treatment with ECT on the test results, these variables were entered into the ANOVAs as covariates separately. This procedure did not alter the statistical results.

The correlation analyses revealed several small to moderate significant associations between depression symptom severity and neurocognitive function. The correlation between MADRS and EMQ was non-significant (Table 4).

A series of similar analyses were run with EMQ and the MCCB test results. None of these correlations reached statistical significance, and there were no discernible non-significant trends in the statistical results (*p*s from .98, TMT-A, to .09, BVMT-R) (data not shown) (see Additional file 1).

## Discussion

To our knowledge, this is the first study of MCCB test performance in relation to depressive symptom level and subjective cognitive complaints in MDD patients.

On all the tests of the MCCB, the patient group was outperformed by the healthy control group. The largest group differences were found for Speed of Processing and Reasoning/Problem Solving. As the test used to assess Reasoning/Problem Solving domain is speed based, our results support other findings of psychomotor speed being strongly affected in depression disorders [1–3, 7, 13, 39].

Regarding the pattern of neurocognitive dysfunction, there are certain differences between our study and other relevant studies in this field. Rund et al. [13] found larger impairments in verbal learning and working memory compared to us. However, they used related, but different tests than we did, as well as more tests per domain.

The MCCB tests responses reported in the Kessler et al. [7] study were highly similar to ours. Therefore, our results suggest that the MCCB is applicable for MDD as well as for bipolar disorders.

According to our cut-off point of 1.5 *SD* below the control mean, at least one fifth of our patients demonstrated neurocognitive scores that indicate clinical levels of impairment in all domains, and in Speed of Processing and Reasoning/Problem Solving, more than half were clinically impaired. These figures are higher than in the Rund et al. [13] study, probably because our patients had been classified as treatment-resistant and thus more severely ill. Moreover, our percentages of impairment are slightly higher than those of the Kessler et al. [7] study, likely due to a combination of divergent number of participants as well as different diagnoses within the depression disorder spectrum.

Our patient group was heterogeneous in terms of the presence of symptoms of bipolar disorder type II and psychosis. Moreover, some of the patients had previously been treated with ECT. However, the statistical control for these variables did not alter the above results.

The severity of depression symptoms was significantly correlated with most of the neurocognitive tests. This contrasts with the findings of Kessler et al. [7], who reported no such associations in bipolar I and II disorder patients. However, the different diagnostic characteristics of these two studies may explain the divergent results. Neither in the Rund et al. [13] study of depressed patients was there any significant relations between symptom load and cognitive test results, although this could be explained by their employment of different assessment methods to ours.

We found no significant relationship between subjectively reported cognitive complaints and MCCB performance. This is in accordance with other reports of such discrepancy [15]. This result may reflect a general tendency for pessimism and self-deprecation in depressed individuals. However, the neuropsychological laboratory tests may lack the ecological validity necessary for tapping experiences of cognitive problems in everyday life [16]. Moreover, the test situation per se is different from one’s private life at work or at home. During neuropsychological assessment, one is usually able to muster the effort and motivation to perform the tests as a function of having volunteered for the research project and being given detailed instructions by the test technician, while there may be little reason for concentration and effort when one is alone at home.

In order to obtain a complete picture of the relationship between subjective complaints and objective test performance, the EMQ should be administered to the control group as well. The fact that this was not done is a limitation of the present study.

### Strenghts and limitations

The major strengths of this study are our pairwise matched samples and the use of a comprehensive, internationally validated neurocognitive test battery with strong psychometric properties.

The major limitation is the relatively small samples. Consequently, we were not able to compare the patients with MDD alone to those with symptoms of bipolar disorder II or psychosis. However, the statistical control we performed indicated that these added factors had no significant effect on neurocognitive function. Moreover, our heterogeneous patient group is a typical naturalistic sample, and our findings are probably clinically valid.

Another limitation is the lack of gender balance, as there were twice as many women than men in our samples. The gender differences in MCCB performance in Norway are not large among healthy respondents [25], but there is a lack of information on possible gender differences in responses among depressed individuals.

Third, the patient sample consisted of hospitalized individuals awaiting ECT for severe treatment-resistant depression. Possibly, our results may not generalize to outpatients or less severely depressed people.

Fourth, several different clinicians were involved in the diagnostic process. The patients were severely ill, and the decision to commence ECT was sometimes made so rapidly that the diagnostic process could not be undertaken by one and the same clinician. We did, however, rely on information from the patients’ journals as well as the diagnostic interviews to ascertain the main diagnosis of MDD.

A final limitation concerns the use of the MCCB. This battery was assembled and validated for schizophrenia spectrum disorders, and may not be the optimal way of assessing cognitive function in MDD. Most of the MCCB studies in non-schizophrenia samples have been conducted with bipolar disorder patients, and although this battery seems applicable in this group [6], it is recommended that more executive function tests are added to the MCCB in order to capture the full extent of cognitive dysfunction in bipolar disorders [9]. In unipolar depression, an internationally validated consensus battery of tests of cognitive deficits is currently lacking. The cognitive deficits in MDD are assumed to be less severe compared to those of bipolar disorders and schizophrenia [40]. Possibly, the MCCB is not sensitive enough to detect all of the more subtle cognitive impairments in this disorder. Moreover, although we obtained distinctive neurocognitive profiles in our depression patients and healthy controls, our samples were small. Our conclusions must be regarded as tentative pending future studies with larger samples.

## Conclusions

The MCCB was able to separate the neurocognitive profile of the current depression patients from that of the healthy controls. There was a general cognitive deficit in MDD patients compared to controls, with at least one fifth of the patients demonstrating impairment across domains. Psychomotor speed functions were most severely affected. This cognitive dysfunction may be partly explained by depression intensity. There were no significant relationship between subjective cognitive complaints and objective neuropsychological test results.

## Ethics

All participants signed an informed consent form before testing. This study was approved by the Regional Committee for Research Ethics for Health Region South-East (REK Sør-Øst).

## Consent to publish

All participants signed and informed consent form agreeing to participation in the project and publication of the results.

## Availability of data and materials

For ethical reasons, the following variables were removed from the currently provided data set: Gender, Psychiatric and Somatic Diagnoses, and Substance abuse. All the analyses (presented in Tables 1, 2, 3, and 4) may be reproduced with this data set. Inquiries about the data may be made to the corresponding author.

## Additional file

Additional file 1: Mohn Rund BMC Psych 2016. (XLSX 15 kb)
